# Higher Medical Education

**Published:** 1893-06

**Authors:** 


### HIGHER MEDICAL EDUCATION.

We are glad to see the medical colleges of the country taking radical steps looking to higher medical education, and sincerely trust that the efforts of the colleges in this direction will be met and aided by proper legislation in this and other States. The public needs the mighty arm of the law to protect it from quacks, charlatans and incompetent practitioners of medicine and surgery. Legal redress in the form of suits for mal-practice, comes too late when life or limb are the questions at issue. Money cannot pay for the mistakes of the Doctor. The legislatures of many Northern States have taken the matter in hand, and these States now have adequate laws regarding the licensing of practitioners of medicine.

The Georgia law says : "No person shall practice medicine within this State unless he has been heretofore legally authorized so to do, or shall be hereafter authorized so to do, by a diploma from an incorporated medical college, medical school or university." The law goes further than this and states that the *Georgia Colleges* shall require attendance upon at least two courses of lectures before granting a diploma. But any one holding a diploma from any regularly chartered medical college or university, can register such diploma in Georgia and obtain a license to practice medicine and surgery within her borders. The college granting such diploma, if not within the State, may have as many or as few courses as it chooses, yet its diploma gives to its holder the right to practice in Georgia, on an equal footing with all.

The point we wish to make is, that the licensing power is vested in the State and not in the colleges, and that however willing or anxious the colleges may be to elevate the standard of medical education in this State, they are powerless unless the law will sustain them.

We hope and believe that the bill framed by the last legislature requiring of applicants for license, not only the registration of a diploma from a college requiring three courses, but also the passage of a satisfactory examination before a State board of medical examiners, will become a law. Not only will the Doctors of the State accept it, but the *people* of Georgia *demand* of the State this much protection at her hands, against ignorance and incompetency in the medical profession.

